# Aberrant Gene Expression in Dogs with Portosystemic Shunts

**DOI:** 10.1371/journal.pone.0057662

**Published:** 2013-02-25

**Authors:** Frank G. van Steenbeek, Lindsay Van den Bossche, Guy C. M. Grinwis, Anne Kummeling, Ingrid H. M. van Gils, Marian J. A. Groot. Koerkamp, Dik van Leenen, Frank C. P. Holstege, Louis C. Penning, Jan Rothuizen, Peter A. J. Leegwater, Bart Spee

**Affiliations:** 1 Department of Clinical Sciences of Companion Animals, Faculty of Veterinary Medicine, Utrecht University, Utrecht, The Netherlands; 2 Department of Pathobiology, Faculty of Veterinary Medicine, Utrecht University, Utrecht, The Netherlands; 3 Molecular Cancer Research, University Medical Centre Utrecht, Utrecht, The Netherlands; University of Modena & Reggio Emilia, Italy

## Abstract

Congenital portosystemic shunts are developmental anomalies of the splanchnic vascular system that cause portal blood to bypass the liver. Large-breed dogs are predisposed for intrahepatic portosystemic shunts (IHPSS) and small-breed dogs for extrahepatic portosystemic shunts (EHPSS). While the phenotype resulting from portal bypass of the liver of the two types of shunt is identical, the genotype and molecular pathways involved are probably different. The aim of this study was to gain insight into the pathways involved in the different types of portosystemic shunting. Microarray analysis of mRNA expression in liver tissue from dogs with EHPSS and IHPSS revealed that the expression of 26 genes was altered in either IHPSS or EHPSS samples compared with that in liver samples from control dogs. Quantitative real-time PCR of these genes in 14 IHPSS, 17 EHPSS, and 8 control liver samples revealed a significant differential expression of *ACBP*, *CCBL1*, *GPC3*, *HAMP*, *PALLD*, *VCAM1,* and *WEE1*. Immunohistochemistry and Western blotting confirmed an increased expression of VCAM1 in IHPSS but its absence in EHPSS, an increased WEE1 expression in IHPSS but not in EHPSS, and a decreased expression of CCBL1 in both shunt types. Regarding their physiologic functions, these findings may indicate a causative role for VCAM1 in IHPSS and WEE1 for IHPSS. CCBL1 could be an interesting candidate to study not yet elucidated aspects in the pathophysiology of hepatic encephalopathy.

## Introduction

Congenital portosystemic shunts (CPSS) are vascular anomalies by which portal blood circumvents the liver, flowing directly into the systemic circulation. As a result, portal blood does not undergo hepatic metabolism through the liver parenchyma [1]–[3]. The associated hepatic dysfunction gives rise to several central nervous system, gastrointestinal tract, and urinary tract symptoms and signs [1], [4], [5]. For example, exposure of the brain to endogenous neurotoxic substances can lead to hepatic encephalopathy [6]. Two anatomically different types of shunt have been described. Intrahepatic portosystemic shunts (IHPSS) are usually embryological shunts (ductus venosus) in the liver that failed to close after birth, whereas extrahepatic shunts (EHPSS) are developmental vascular anomalies by which the extrahepatic portal system is connected with the caudal vena cava or (hemi)azygos vein [7]. The functional consequences, virtual absence of portal perfusion of the liver parenchyma, and clinical signs are identical for both types of shunt [8], [9].

CPSS occur sporadically in a variety of species, including humans [10], but frequently in dogs (*Canis lupus familiaris*). There are no essential differences between humans and dogs with CPSS with regard to the histological features of the liver, clinical presentation, and diagnostic methods [7]. Excessive inbreeding of purebred dog populations has greatly increased the incidence of genetic disorders [7] and genetic association analyses in specific dog breeds have shown that canine model systems can provide unique insights into human biology and disease [11], [12]. CPSS are mainly found in purebred dogs [9], [13]–[15] and, in general, IHPSS are typically seen in large-breed dogs such as Irish wolfhounds [16], Golden retrievers [17], Labrador retrievers [17], [18], Australian cattle dogs [19], and Old English sheepdogs [5]. EHPSS occur in small-breed dogs such as Cairn terriers [9], Yorkshire terriers [15], [17], Jack Russell terriers [20] Dachshunds [18], Miniature schnauzers [17], and Maltese terriers [19]. In evaluated dog breeds, IHPSS [8], [14], [21] and EHPSS [9], [14], [15] proved to be inheritable disorders. Test matings and pedigree analysis of Irish wolfhounds has shown that IHPSS are not a monogenetic trait but possibly caused by two interacting genes [8]. Similar analyses in Cairn terriers have indicated that the genetic basis of EHPSS is more complex and does not follow simple Mendelian rules of inheritance [9]. The confirmation that portosystemic shunting has a genetic basis in these breeds makes the dog an ideal model with which to unravel the embryonic development of the ductus venosus and the intrahepatic and extrahepatic portal system.

Progressive liver disease is an ailment common to both humans and dogs, and the regulatory pathways involved in chronic fibrotic liver disease, which ultimately leads to liver cirrhosis, are the same in both species [22]–[24]. Impaired hepatic perfusion plays an important part in the chronic deterioration of liver function seen in progressive liver disease [25]–[27]. Knowledge of the genes and metabolic pathways implicated in CPSS might provide insight into the pathways involved in the vascular derangements of chronic progressive liver diseases, which in turn might lead to new ways to intervene in these currently incurable diseases [7].

In the present experiment, RNA samples isolated from the liver of dogs with IHPSS and EHPSS were used for gene profiling, and differential gene expression was confirmed by qPCR and immunohistochemistry. We demonstrate aberrant expression of certain genes in dogs with all types of CPSS attributed to the shared phenotype. In addition, few genes were differentially expressed between dogs with EHPSS or IHPSS, implying genotypic differences involved in these pathophysiologically comparable liver diseases.

## Materials and Methods

### Animals

Control tissue was obtained from six healthy mature dogs sacrificed for unrelated studies. The absence of underlying liver disease in these dogs was confirmed histologically by a board certified veterinary pathologist. Dogs with CPSS were kept privately as companion animals and were presented to the University Clinic for Companion Animals (Department of Clinical Sciences of Companion Animals, Utrecht University), where CPSS was diagnosed on the basis of increased fasting plasma levels of ammonia [8], [9] and ultrasound visualization and classification of shunts. All affected dogs underwent surgery, during which the diagnosis and classification were confirmed. Wedge biopsies of the liver were taken during surgical closure of the shunt, and effects of portal hypoperfusion were identified histologically in all animals, a finding that is consistent with CPSS [18]. Before and 2 months after surgery, the size of the liver was assessed by ultrasound, and the extent of portosystemic shunting of portal blood was assessed with a rectal ammonia tolerance test and Doppler ultrasound of the original shunt. Ten dogs with EHPSS made a complete recovery, based on normalization of liver size and the absence of flow in the shunting vessel; a second liver biopsy was then taken from these animals. Liver samples were snap frozen in liquid nitrogen or RNAlater (Ambion, Inc., Austin, Texas) for RNA isolation, or fixed in 10% neutral buffered formalin and embedded in paraffin for immunohistochemistry. Since some of the samples were obtained at necropsy and others at biopsy, tissue fixation times and the ratio of tissue volume: fixative volume varied between animals, which could influence staining. The procedures were approved by the local ethics committee, as required under Dutch legislation (ID 2007.III.08.110).

### Expression profiling

Total RNA was isolated from liver tissue from 2 healthy dogs, 32 dogs with EHPSS, and 15 dogs with IHPSS (Table 1), using a RNeasy Mini Kit (Qiagen, Venlo, the Netherlands) and on-column DNase digestion. RNA quality and quantity was determined on a nanochip (Bioanalyzer, Agilent Technologies, Santa Clara, US). RIN values above 8.0 were considered reliable, and these samples were included in the study. Pooled RNA isolated from healthy liver tissue was used as reference. Agilent Canine Gene Expression Microarray V1 containing 42,034 60-mer probes in a 4×44K layout was used to determine genome wide expression, using 3 μg of total RNA from each animal co- hybridized to the common reference. RNA amplification and labeling were performed [28] on an automated system (Caliper Life Sciences NV/SA, Belgium). Dye swap of Cy3 and Cy5 was performed to reduce dye bias. Hybridization was done on a HS4800PRO system supplemented with QuadChambers (Tecan Benelux B.V.B.A.), using 1 µg labeled cRNA per channel [29].

**Table 1:** Samples from dogs with extrahepatic portosystemic shunts (EHPSS) or intrahepatic portosystemic shunts (IHPSS) used for microarray or qualitative PCR analysis.

		microarray		qPCR		Postoperative confirmation	
	status	*female*	*male*	*female*	*male*	*female*	*male*
Cairn terrier	EHPSS	3	4	2	4	2	1
Cross breed	EHPSS	2	1	0	0	2	1
Jack Russell terrier	EHPSS	3	3	2	1	1	0
Maltese terrier	EHPSS	3	2	2	1	0	0
Miniature dachshund	EHPSS	1	0	0	0	0	0
Norfolk terrier	EHPSS	2	1	2	0	0	0
Shih Tzu	EHPSS	1	0	0	0	1	0
West Highland white terrier	EHPSS	2	0	1	0	1	0
Yorkshire terrier	EHPSS	4	0	1	0	0	0
Australian shepherd	IHPSS	1	0	1	0	0	0
Bearded collie	IHPSS	0	1	0	1	0	0
Bernese mountain dog	IHPSS	2	1	2	1	0	0
Cane corso	IHPSS	0	1	0	1	0	0
Duck tolling retriever	IHPSS	0	1	0	1	0	0
Golden retriever	IHPSS	2	1	2	1	0	0
Hovawart	IHPSS	0	1	0	1	0	0
Irish wolfhound	IHPSS	2	0	1	0	0	0
Labrador retriever	IHPSS	0	1	0	1	0	0
Newfoundland	IHPSS	1	0	1	0	0	0

Hybridized slides were scanned on an Agilent scanner (G2565BA) at 100% laser power, 30% photomultiplier tube voltage, and automated data extraction was done using Imagene 8.0 (BioDiscovery). Normalization was performed with Loess [30] on mean spot intensity, and dye bias was corrected based on a within-set estimate [31].

group and the control (healthy liver). Data were analyzed using ANOVA (R version 2.2.1/MAANOVA version 0.98–7) [32]. Correction for multiple testing (Permutation F2-test using 5,000 permutations) was performed and P<0.05 was considered statistically significant. Genes with log2-fold changes of more than 0.4 or less than −0.4 were then selected to ensure that only robust changes were considered.

### Amplification for qPCR

Liver samples (16 from dogs with EHPSS and 14 from dogs with IHPSS) were randomly selected for confirmation by qPCR after RNA amplification with the WT-Ovation RNA Amplification System (Bemmel, the Netherlands), using 80 ng RNA per sample. This system converts RNA to cDNA, using the linear isothermal DNA amplification called SPIA [33], which produces single-strand DNA. The products were diluted three times and stored at −20°C until used. To match experimental conditions, RNA from control samples was treated in a similar fashion and a water sample was used as a negative control.

### qPCR

Perlprimer v1.1.14 was used for primer design on Ensembl annotated transcripts and the amplicon was tested for secondary structures using MFold [34]. Gradient PCRs were performed to determine the optimum temperature for obtaining 100% PCR efficiency. Primer specificity was validated *in silico* (BLAST specificity analysis) and empirically (DNA sequencing, gel electrophoresis, and melting profiles). qPCR reactions were performed in 25-µl duplicates containing 0.5× SYBR Green-Supermix (BioRad, Veenendaal, the Netherlands), 0.4 µM primer, and 1 µl cDNA. Five reference genes were used for normalization, based on their stable expression in liver, namely, *glyceraldehyde-3-phosphate dehydrogenase (GAPDH), β-2-microglobulin (B2M), ribosomal protein S5 (RPS5), heterogeneous nuclear ribonucleoprotein H (HNRPH),* and *ribosomal protein S19 (RPS19)* [35]. GeneNorm [36] was used to establish stability. Primers for reference genes and genes of interest, including their optimum temperature, are listed in table 2. Cycling conditions were a 3-minute Taq polymerase activation step at 95°C, followed by 45 cycles of 10 seconds at 95°C for denaturation, and 30 seconds at T_m_ for annealing and elongation. All experiments were conducted with a MyiQ Single-Color Real-Time PCR Detection System (BioRad). A 4-fold standard dilution series of a pool containing all samples was used to determine relative expression. Data analysis was performed with IQ5 Real-Time PCR detection system software (BioRad). Expression levels were normalized by using the average relative amount of the reference genes. Log-values of normalized relative expression were used to obtain normal distribution. A Wilcoxon rank sum test was performed in R [37] to determine the significance of differential gene expression.

**Table 2:** Primers used for qualitative PCR.

Gene	Ensembl TranscriptID	F/R	sequence	T_m_(°C)	Amplicon Size (bp)
B2M	ENSCAFT00000038092	F	5′-TCCTCATCCTCCTCGCT-3′	61.2	85
		R	5′-TTCTCTGCTGGGTGTCG-3′		
GAPDH	ENSCAFT00000037560	F	5′-TGTCCCCACCCCCAATGTATC-3′	58	100
		R	5′-CTCCGATGCCTGCTTCACTACCTT-3′		
HNRPH	ENSCAFT00000028063	F	5′-CTCACTATGATCCACCACG-3′	61.2	151
		R	5′-TAGCCTCCATAACCTCCAC-3′		
RPS19	ENSCAFT00000008009	F	5′-CCTTCCTCAAAAAGTCTGGG-3′	61	95
		R	5′-GTTCTCATCGTAGGGAGCAAG-3′		
RPS5	ENSCAFT00000003710	F	5′-TCACTGGTGAGAACCCCCT-3′	62.5	141
		R	5′-CCTGATTCACACGGCGTAG-3′		
ABCC11	ENSCAFT00000016007	F	5′-AAGTTCTCCATTGTCCCTC-3′	57.7	90
		R	5′-TCTGTTCATCTGTGTAACGA-3′		
ACBP	ENSCAFT00000007872	F	5′-GTTAAGCACCTCAAGACCA-3′	64.1	96
		R	5′-GCCGTTCTGTGTTTATGTC-3′		
APOA1	ENSCAFT00000021138	F	5′-CAGTCAAAGACAGCGGCAG-3′	61.2	166
		R	5′-CTCCAGGTTATCCCAGAACTCC-3′		
BCHE	ENSCAFT00000023011	F	5′-CTCAACAATGCCGATTCTG-3′	56	84
		R	5′-CTCCATTCTCGTTCTGCT-3′		
BRP44	ENSCAFT00000024369	F	5′-GCTGTTAATTTCTTTGTGGGTG-3′	63.7	110
		R	5′-TCAGGTGGTCAGGAACTC-3′		
CAPS	ENSCAFT00000029761	F	5′-AGTAGGACAAAGGTTCCGA-3′	59.3	197
		R	5′-GCAATCTCAAGTGGTGGG-3′		
CCBL1	ENSCAFT00000031874	F	5′-CATCGCAGACATCTCAGAC-3′	58.7	182
		R	5′-AAACAGAAGCGGATATAGTGG-3′		
CYP2E1	ENSCAFT00000021134	F	5′-GTAGCAAACCAGGACACGA-3′	65.7	247
		R	5′-GCGGACAAGAACAGGAAGAG-3′		
DSTN	ENSCAFT00000008828	F	5′-GCACCAGAACTAGCTCCT-3′	64	200
		R	5′-GCACTGAATGATGGTCTACAC-3′		
GATM	ENSCAFT00000021782	F	5′-CTCCTCCAATACCAGTCATCC-3′	58.8	219
		R	5′-ACATCACAGGTCCAGCAG-3′		
GDF15	ENSCAFT00000023627	F	5′-CTGGTGATACTGGTGATGCT-3′	66.8	202
		R	5′-AGGTCAGGGTTTGAATCGG-3′		
GPC3	ENSCAFT00000029940	F	5′-AGAAGAATGGTGGAAAGCTGAC-3′	68.1	138
		R	5′-CTATACTGGCGTTGTTGAGAATGG-3′		
HAMP	ENSCAFT00000011304	F	5′-CCAGTGTCTCAGTCCTTCC-3′	65.5	163
		R	5′-TTTACAGCAGCCACAGCA-3′		
JDP2	ENSCAFT00000026985	F	5′-CTGAAATACGCCGACATCC-3′	61.1	153
		R	5′-CCGCCACTTTGTTCTTCTC-3′		
KIFC2	ENSCAFT00000002564	F	5′-CCATCTCAAGAAGAAAGCCC-3′	60.7	246
		R	5′-GTTTCAGAGCCTCATTCTCC-3′		
MPND	ENSCAFT00000030318	F	5′-GGCTTCTGTCAAGTACAAGGG-3′	65.7	142
		R	5′-CTTCCTCCATCAACAGCTCCT-3		
PALLD	ENSCAFT00000012001	F	5′-GTTAAGCACCTCAAGACCA-3′	62.7	96
		R	5′-GCCGTTCTGTGTTTATGTC-3′		
PON3	ENSCAFT00000003345	F	5′-AGAACTGCCGCCTTATTGAG-3′	62.1	241
		R	5′-GATGAAAGTACTGATTCCGTGTG-3′		
SERPINA7	ENSCAFT00000028383	F	5′-GACCTCAAACCAAACACCA-3′	62.2	101
		R	5′-GCTGAAACCCTCTTCTGTC-3′		
SLC1A2	ENSCAFT00000011054	F	5′-ACCATGCTCCTCATCCTG-3′	63.7	102
		R	5′-CATTGACTGAAGTTCTCATCCT-3′		
VCAM1_1	ENSCAFT00000031837	F	5′-GATGAAATTGACTTTGAGCCCA-3′	65	127
		R	5′-ATTGTCACAGAACCGCCT-3′		
VCAM1_2	ENSCAFT00000031837	F	5′-AGTTAGAGGATGCGGGAG-3′	63	132
		R	5′-TAAAGCACGAGTAGTTCTGG-3′		
WEE1	ENSCAFT00000011883	F	5′-AGAGGCAGAGTTGAAGGA-3′	65	130
		R	5′-CAGCATTTGGGATTGAGGT-3′		
ZCCHC9	ENSCAFT00000013818	F	5′-ACAGTCAGGAGGTAAGGG-3′	63.2	197
		R	5′-CACAGCGATAACATATTCCAG-3′		

B2M = β-2-Microglobulin, GAPDH = Glyceraldehyde-3-phosphatedehydrogenase, HNRPH = Heterogeneous nuclear ribonucleoprotein H, RPS19 = Ribosomal protein S19, RPS5 = Ribosomal protein S5, ABC11 =  ATP-binding cassette, sub-family C (CFTR/MRP), member 11, ACBP =  Diazepam binding inhibitor (GABA receptor modulator, acyl-CoA binding protein), AFM = afamin, APOA1 = Apolipoprotein A-I, BCHE = Butyrylcholinesterase, BRP44 = Brain protein 44, CAPS =  Calcyphosine, CCBL1 = Cysteine conjugate-beta lyase, cytoplasmic, cOR13P3 = cOR13P3 olfactory receptor family 13 subfamily P-like, CYP2E1 = Cytochrome p450 2E1, DSTN = Destrin (actin depolymerizing factor), GATM = Glycine amidinotransferase, GDF15 = Growth differentiation factor 15, GPC3 = Glypican 3, HAMP = Hepcidin antimicrobial peptide, JDP2 = Jun dimerization protein 2-like, KIFC2 = Kinesin family member C2, MPND = MPN domain containing, PALLD = Palladin, cytoskeletal associated protein, PON3 = Paraoxonase 3, SERPINA7 = Serpin peptidase inhibitor, clade A (alpha-1 antiproteinase, antitrypsin), member 7, SFTPD = surfactant protein D, SLC1A2 = Solute carrier family 1 (glial high affinity glutamate transporter), member 2, VCAM1 = Vascular cell adhesion molecule 1, WEE1 = WEE1 homolog (S. pombe), ZCCHC9 = Zinc finger, CCHC domain containing 9).

### Immunohistochemistry

Liver samples from healthy dogs (n = 6) and randomly selected dogs with IHPSS (n = 6) and dogs with EHPSS (n = 6) were stained for ACBP, CCBL1, HAMP, GPC3, PALLD, VCAM1, and WEE1, using reagents and methods described in Table 3. Five-micrometer sections of paraffin-embedded liver tissue were deparaffinized in xylene, and rehydrated in an ethanol to water series.

**Table 3:** Antibody specifications.

Primary Antibody	Manufacturer	Catalogue no.	Dilution IHC	Diluent	Incubation time	Antigen retrieval	Type sec. AB	Incubation DAB (min)	Dilution WB
ACBP	Abnova	mab0725	1:200	PBS+BSA	O/N 4°C	TE-buffer, 40 min	mouse monoclonal	2	
CCBL1	Sigma	hpa021177	1:500	ABD	O/N 4°C	Proteinase K, 10 min	rabbit polyclonal	2	1:1000
HAMP	Abcam	ab30760	1:200	ABD	1h RT	Proteinase K, 10 min	rabbit polyclonal	2	
GPC3	BioMosaics	B0025R, B0055R	1:50	ABD	O/N 4°C	TE-buffer, 30 min	mouse monoclonal	4.5	
PALLD	Novus	NBP1-25959G	1:25	PBS+BSA	O/N 4°C	Citrate-buffer 40 min	mouse monoclonal	5	
VCAM1	Santa Cruz	sc-8304	1:100	PBS	O/N 4°C	Proteinase K, 10 min	rabbit poloclonal	1	1:500
WEE1	Santa Cruz	sc-5285	1:50	PBS	O/N 4°C	TE-buffer, 40 min	mouse monoclonal	5	1:20
ACTB	Thermo Fisher Scientific						mouse		1:2000

IHC  =  immunohistochemistry, WB  =  Western Blot, ABD  =  antibody diluent (DAKO), PBS =  Phosphate-buffered saline, BSA = Bovine serum albumin, TE = Tris-Ethylenediaminetetraacetic acid, ACTB  =  β-actin.

Heat-induced antigen retrieval was performed with 10 mM citrate buffer (pH 6.0) or 10 mM Tris with 1 mM EDTA (pH 8.0) at 98°C in a water bath, followed by cooling at room temperature (RT) for 30 minutes (Table 3). Antigen retrieval by enzymatic digestion was performed with proteinase K (Dakocytomation, Glostrup, Denmark) for 10 minutes at RT. Dual endogenous enzyme block (Dakocytomation) was used (10 minutes RT) to quench endogenous peroxidase activity, and background staining was blocked with 10% normal goat serum (Sigma-Aldrich, St. Louis, US) (30 minutes). Sections were incubated with the labeled secondary antibody Envision (Dakocytomation) for 1 hour at RT. The signal was developed in 0.06% 3,3′-diaminobenzidine (DAB) solution (Dakocytomation) for the indicated time (Table 3) and counterstained with Hematoxylin QS (Vector Laboratories, Burlingame, US). Replacement of primary antibody with washing buffer served as negative control. All tissues were stained in batch per antibody to avoid technical differences.

All immunohistochemically stained sections were evaluated by a board-certified pathologist (GCMG) who was unaware of the dogs' phenotype, using a semi-quantitative scoring system based on the intensity and localization of staining, with grading as follows: 0, absent; 1, mild positive staining; 2, moderate positive staining; 3, strong positive staining. If different histological elements (hepatocytes, bile ducts, Kupffer cells) were stained, then staining in these elements was scored separately. Information on acinar localization (zone 1, 2, or 3) was also collected. The average staining intensity score for each group (i.e. intrahepatic, extrahepatic, control) was calculated. Student's t-test was used to detect significant differences in staining intensity, with P<0.05 being considered statistically significant.

### Western blot

For Western blot analysis 30 mg of liver tissue from at least four samples of each group (healthy n = 4, IHPSS n = 4, EHPSS n = 4, randomly chosen from original group) were homogenized in RIPA buffer (Sigma-Aldrich). Protein concentrations were obtained using a Lowry-based assay (DC Protein Assay, BioRad). 30 μg of protein of the supernatant was denatured for 2 min at 95°C and separated on 7.5% (VCAM1 and CCBL1) or 10% (WEE1) Tris-HCl Criterion gels (BioRad) and the proteins were transferred onto Hybond-C Extra Nitrocellulose membranes (Amersham Biosciences Europe, Roosendaal, The Netherlands). The membranes were incubated with 4% non-fat dry-milk (BioRad) in TBS for 1 hour with shaking. The incubation of the primary antibody was performed at 4°C over-night for all antibodies (see Table 3) in TBS with 0.1% Tween-20 (Boom B.V., Meppel, The Netherlands) and 4% Bovine Serum Albumin (BSA). After washing, the membranes were incubated with their respective horseradish peroxidase-conjugated secondary antibody (R&D systems, Europe Ltd., Abingdon, UK) at room temperature for 1 h. Immunodetection was performed with an ECL Western blot analysis system, performed according to the manufacturer's instructions (BioRad). Replacement of primary antibody with TBST and 4% BSA served as negative control. β-Actin (ACTB) was used as loading control. Imaging was performed on a ChemiDoc XRS System (BioRad) and the intensity of the bands was quantified using Quantity One 4.3.0 Software (BioRad).

## Results

### Expression profiling

**Figure 1:**
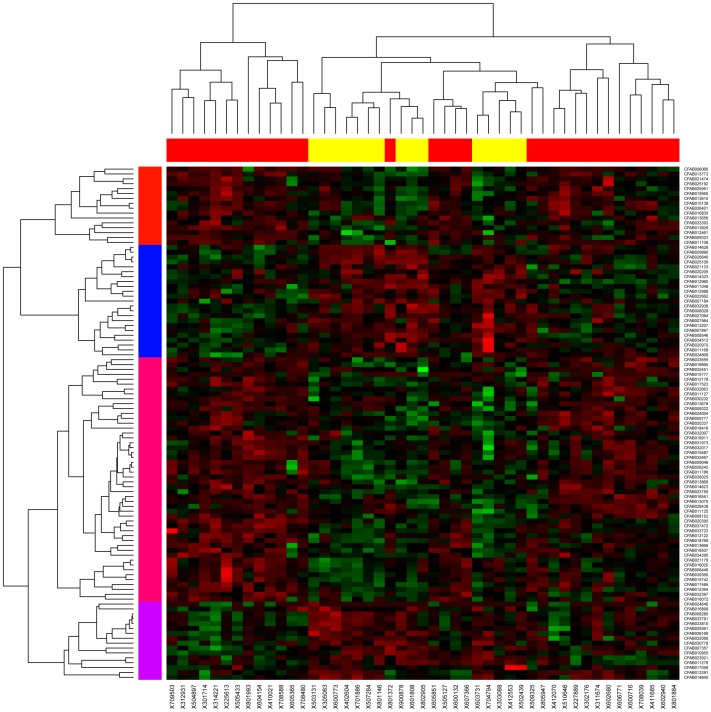
Heatmap EHPSS vs IHPSS. 107 annotated probes (listed in rows) were expressed significantly differently in the 32 dogs with extrahepatic portosystemic shunts (EHPSS; red columns) and 15 dogs with intrahepatic portosystemic shunts (IHPSS; yellow columns) compared with control dogs.

The expression of 142 probes was significantly different compared to the controls in samples from dogs with EHPSS or IHPSS (Figure 1), of which only 107 were annotated (CanFam 3.1). Of these, 19 and 6 annotated genes were specific to liver samples from dogs with either IHPSS or EHPSS, respectively (Table 4). Additionally, *HAMP* was significantly downregulated in dogs with IHPSS and significantly upregulated in dogs with EHPSS compared with healthy dogs (Table 4). The other 81 annotated genes were up- or downregulated in both groups of dogs, often more strongly in one phenotype than in the other. To avoid analyzing secondary effects, these genes were excluded. All data have been deposited in NCBI's Gene Expression Omnibus [38] and are accessible through GEO Series accession number GSE39005 (http://www.ncbi.nlm.nih.gov/geo/query/acc.cgi?acc=GSE39005).

**Table 4:** Genes expressed differently in dogs with or without extrahepatic (EHPSS) or intrahepatic (IPHSS) portosystemic shunts (microarray results in log2).

Gene	IHPSS vs control	EHPSS vs control
ABCC11		0.9
ACBP	-0.8	
AFM	0.9	
APOA1	0.5	
BCHE	0.7	
BRP44	1	
CAPS		−1.3
CCBL1	−0.5	
cOR13P3	0.5	
CYP2E1		0.6
DSTN		−0.5
GATM	0.9	
GDF15	−0.8	
GPC3	1	
HAMP	−0.8	0.7
JDP2	0.5	
KIFC2	0.5	
MPND	0.6	
PALLD		−0.5
PON3	0.5	
SERPINA7	0.9	
SFTPD	−0.7	
SLC1A2		0.7
VCAM1	0.6	
WEE1	0.7	
ZCCHC9	0.6	

### qPCR

**Figure 2:**
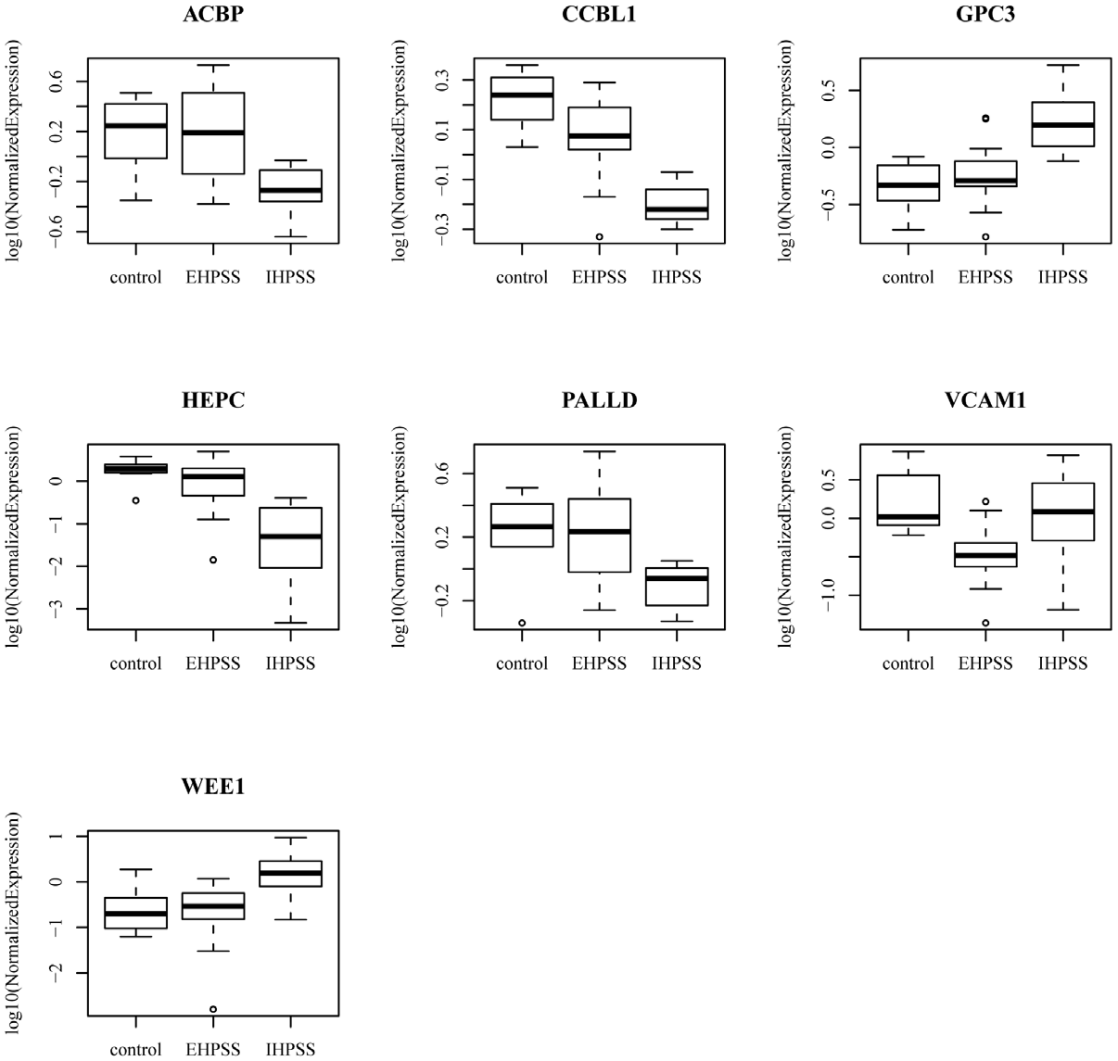
Quantitative PCR results. The upregulation or downregulation of selected genes in liver samples from dogs with or without extrahepatic (EHPSS) or intrahepatic (IHPSS) portosystemic shunts. The thick black line represents the median (50th percentile), also the first and third quartile (25th and 75th percentile respectively) are displayed. Outliers are depicted with an open dot, representing values higher than 1.5 times the interquartile range.

The expression of 23 of the genes differentially expressed in dogs with IHPSS or EHPSS (Table 4) was measured by quantitative RT-PCR. For technical reasons, no qPCR data could be obtained for *AFM*, *SFTPD,* and *cOR13P3*. Only seven genes proved to be differentially expressed in one shunt group (IHPSS or EHPSS) compared with the other shunt group and the healthy controls (Table 5, Figure 2). *ACBP*, *CCBL1*, *HAMP,* and *PALLD* were downregulated (−2.4 to −16.8 fold change) and *GPC3* and *WEE1* (3.8 and 5.1 fold change, respectively) were upregulated in dogs with IHPSS compared with dogs with EHPSS and control dogs. *VCAM1* (−5.5 fold change) was downregulated in dogs with EHPSS compared with dogs with IHPSS and control dogs. These seven genes were not functionally related, based on Metacore^TM^ analysis (GeneGo, St. Joseph, US).

**Table 5:** Genes expressed differently in dogs with or without extrahepatic (EHPSS) or intrahepatic (IPHSS) portosystemic shunts (qPCR results).

	P-value T-test	Bonferroni	Fold change
***ACBP***			
IHPSS vs EHPSS	0.001	0.002	
CONTROL vs EHPSS	0.916	1	
CONTROL vs IHPSS	0.004	0.011	−3.1
***CCBL1***			
IHPSS vs EHPSS	<0.001	<0.001	
CONTROL vs EHPSS	0.021	0.062	
CONTROL vs IHPSS	<0.001	<0.001	−2.7
***GPC3***			
IHPSS vs EHPSS	<0.001	0.001	
CONTROL vs EHPSS	0.427	1	
CONTROL vs IHPSS	<0.001	<0.001	3.8
***HAMP***			
IHPSS vs EHPSS	<0.001	0.001	
CONTROL vs EHPSS	0.154	0.461	
CONTROL vs IHPSS	<0.001	<0.001	−16.8
***PALLD***			
IHPSS vs EHPSS	0.002	0.005	
CONTROL vs EHPSS	0.969	1	
CONTROL vs IHPSS	0.009	0.027	−2.4
***VCAM1***			
IHPSS vs EHPSS	0.014	0.043	
CONTROL vs EHPSS	0.004	0.013	−5.5
CONTROL vs IHPSS	0.435	1	
***WEE1***			
IHPSS vs EHPSS	0.004	0.012	
CONTROL vs EHPSS	0.866	1	
CONTROL vs IHPSS	0.009	0.028	5.1

Relative mRNA expression of the seven differentially expressed genes in qPCR.

**Figure 3:**
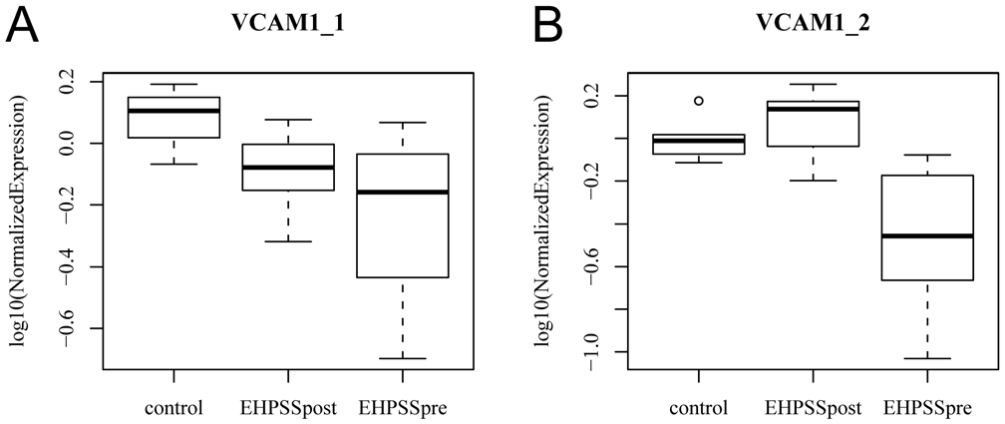
Relative expression of VCAM1 in intraoperative and postoperative samples. Relative expression of VCAM1 mRNA in liver samples from dogs with extrahepatic portosystemic shunts (EHPSS) obtained during and after surgery compared to healthy liver tissue. Samples from postoperative tissue were obtained after EHPSS closure. VCAM1_1 was designed near the 5`-end, VCAM1_2 is located on the 3′-end.

*VCAM1* expression was studied in liver samples taken during and after surgery and compared with that in control liver samples. *VCAM1* expression in liver samples taken during (P = 0.020) and after (P = 0.034) surgery was significantly different from that in control liver samples, but not between the pre- and postoperative liver samples (P = 0.26) (Figure 3A). A second qPCR probe, involving the C-terminus of VCAM1 near the position of the probe for microarray (primer VCAM1_2 table), revealed downregulation of *VCAM1* in liver samples taken during surgery, but not in samples taken after surgery or in control samples (Figure 3B).

### Immunohistochemistry

The intensity of staining for CCBL1, VCAM1, and WEE1 in hepatocytes was significantly different between the two CPSS groups and the control group (Table 6). There were no significant differences in ACBP, GPC3, HAMP, and PALLD staining intensity in the hepatocytes or biliary epithelium.

**Table 6:** Immunohistochemical staining for different proteins in liver samples from dogs with or without extrahepatic (EPHSS) or intrahepatic (IPHSS) portosystemic shunts.

	ACBP		CCBL1		GPC3		HAMP		PALLD		VCAM1		WEE1	
	Mean	P-value	Mean	P-value	Mean	P-value	Mean	P-value	Mean	P-value	Mean	P-value	Mean	P-value
**Control**	2.3		2.8		1.2		1.0		1.8		0.3		0.8	
**EHPSS**	2.7	0.290	1.5	0.006	0.8	0.188	0.3	0.207	1.3	0.209	0.3	0.807	1.7	0.096
**IHPSS**	2.5	0.599	1.0	<0.001	1.0	0.341	0.3	0.145	1.8	1.000	1.8	0.006	1.8	0.044

The mean of the specific protein intensity is listed in the table based on semi-quantitative evaluation of immunohistochemically stained liver biopsies. The corresponding P-value compared to the control group is noticed.

**Figure 4:**
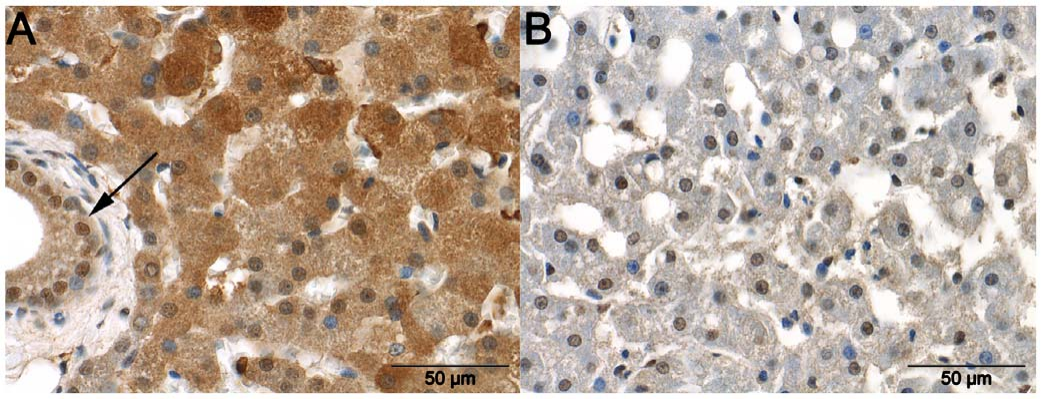
Staining for CCBL1 in the liver. Cysteine conjugate-beta lyase-1 (CCBL1) immunoreactivity in a liver sample from a healthy dog (Figure 4 A) and a dog with an intrahepatic portosystemic shunt (IHPPS) (Figure 4 B). Marked cytoplasmic and moderate nuclear immunoreactivity is visible in hepatocytes and bile duct epithelium (arrow) in the sample from the healthy animal. The sample from the dog with an IHPSS shows only weak immunoreactivity in the cytoplasm and moderate nuclear immunoreactivity of hepatocytes.

CCBL1 staining was typically detected in the cytoplasm (Figure 4), and was more intense in the control dogs than in dogs with EHPSS (P = 0.006) or IHPSS (P = 6.59*10^−7^). Staining was not significantly different between the dogs with IHPSS or EHPSS, although staining was considered more positive in samples from dogs with EHPSS. In some EHPSS cases Kupffer cells also showed a positive staining.

**Figure 5:**
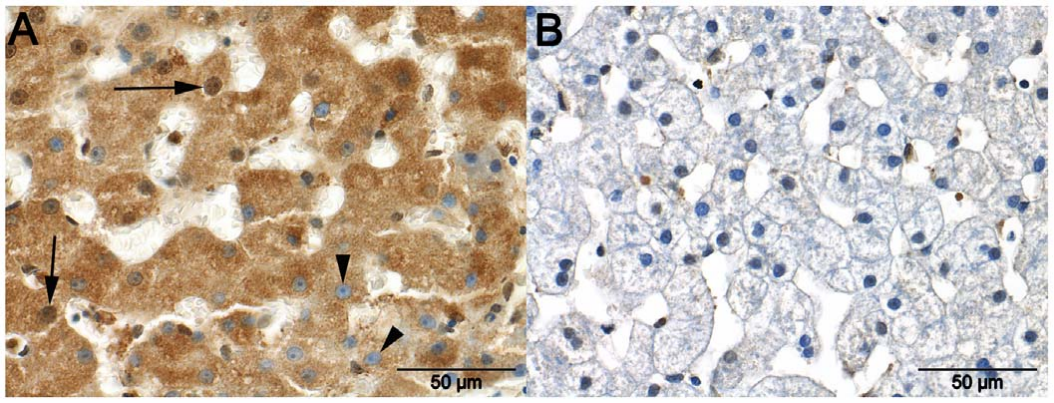
Staining for VCAM1 in the liver. Marked granular cytoplasmic immunoreactivity with the presence (arrows) and absence (arrowheads) of immunoreactivity in the nuclei of hepatocytes in a liver sample taken from a dog with an intrahepatic portosystemic shunt (Figure 5 A). The cytoplasm of hepatocytes in a liver sample from a dog with an extrahepatic portosystemic shunt (EHPPS) show no immunoreactivity. Nuclei in this liver occasionally demonstrate weak immunoreactivity (Figure 5 B).

VCAM1 staining of the cytoplasm and nuclei of samples from control dogs and dogs with EHPSS was mainly negative or moderate in intensity (Figure 5), whereas staining was significantly more intense in samples from dogs with IHPSS than in samples from control dogs (P = 0.006). In addition, all Kupffer cells showed some staining for VCAM1, but no differences were observed between the CPSS and control dogs. Staining of smooth muscle cells was observed around a few blood vessels in most healthy tissues.

**Figure 6:**
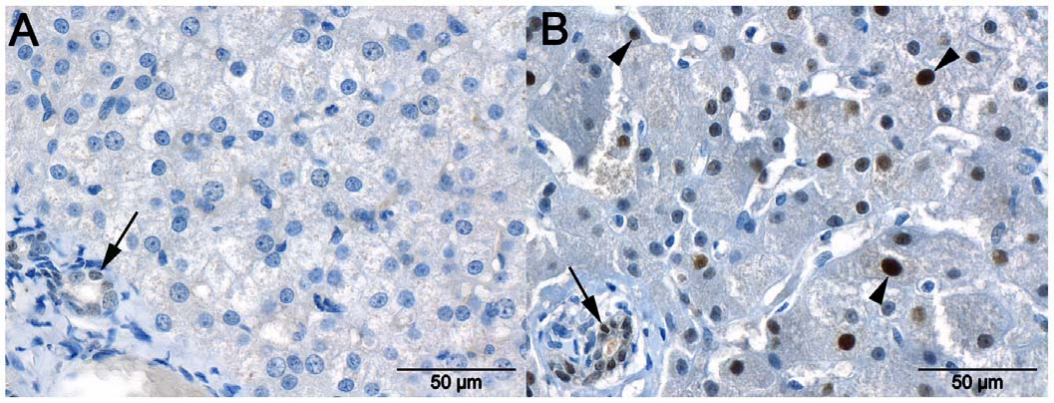
Staining for WEE1 in the liver. Staining for WEE1 in a liver sample from a healthy dog (Figure 6 A) and a dog with an intrahepatic portosystemic shunt (IHPPS) (Figure 6 B). Note the marked nuclear staining in hepatocytes (arrowheads) and bile duct epithelium (arrows) in the sample from a dog with an IHPPS, whereas nuclei of the sample from the healthy dog show only weak staining in bile ducts and no staining in hepatocytes.

WEE1 staining was generally not detected in nuclei (Figure 6), although randomly a few nuclei showed moderate staining. Nuclear staining for WEE1 was found in most bile duct epithelial cells. Nuclear WEE1 staining of hepatocytes was more intense in samples from dogs with IHPSS than in samples from control dogs (P = 0.044), but there were no significant differences in bile duct staining between the three groups of samples.

### Western blot analysis

**Figure 7:**
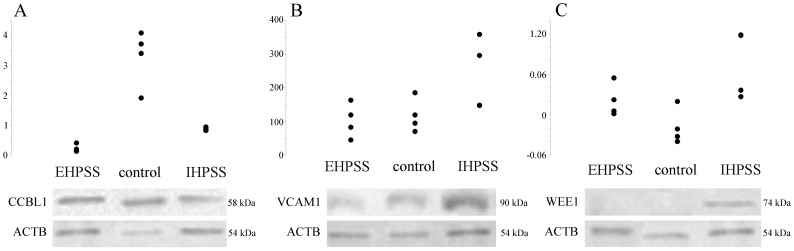
Western blot analyses for CCBL1, VCAM1 and WEE1. Protein expression was measured for CCBL1, VCAM1 and WEE1 in liver tissue of healthy individuals (n = 4) and dogs affected with IHPSS (n = 4) and EHPSS (n = 4). ACTB was used as loading control and replacing primary antibody served as a negative control. CCBL1 was significantly down regulated in both IHPSS as well as EHPSS samples compared to the healthy controls (A). Expression of VCAM1 confirmed the findings of the immunohistochemistry with a downregulation in EHPSS samples was found compared to the IHPSS samples (B). WEE1 was found to be upregulated in IHPSS samples compared to healthy and EHPSS samples (C). The depicted bands are representative for the indicated groups.

Measurement of CCBL1, VCAM1 and WEE1 protein levels in liver samples by Western blotting confirmed the expression differences detected by immunohistochemistry. CCBL1 was significantly downregulated in EHPSS (P = 0.007) and IHPSS (P = 0.002) samples compared to the healthy control tissue (Figure 7A). For VCAM1 an upregulation (P = 0.01) was found in IHPSS samples compared to the two other groups. No differences were found between EHPSS samples and the healthy control group (Figure 7B). Expression of WEE1 was found to be upregulated (P = 0.01) in IHPSS samples compared to the control and EHPSS samples (Figure 7C).

## Discussion

This study used expression profiling to identify pathways involved in the pathogenesis of IHPSS and EHPSS. Both types of shunt give rise to impaired portal perfusion of the liver parenchyma, which results in decreased growth, liver dysfunction, and clinical symptoms. However, IHPSS are typically seen in large-breed dogs and EHPSS are typically seen in small-breed dogs [7], which suggests that the causative genotype is most likely different. Genes possibly involved in a specific type of shunt were identified by comparing gene expression in liver sample from dogs with IHPSS or EHPSS, and control dogs. Differences in the hepatic expression of genes in dogs with IHPSS or EPHSS were interpreted as indicating specific characteristics of each subtype, whereas differences shared by dogs with IHPSS or EHPSS compared with controls dogs are most likely due to secondary effects, such as the absence of normal portal vein perfusion of the liver. The main differences in mRNA gene expression were further evaluated at the protein level. On the basis of quantitative differences in both RNA and protein expression, VCAM1 may be associated with the phenotype of EHPSS, and with that of IHPSS. Functional analysis will be needed to evaluate the precise role of these genes in dogs with CPSS.

Genes of interest were initially selected on the basis of microarray analysis; about 40% of the probes on the array have not yet been annotated (CanFam 3.1). Of the 142 probes that were expressed differently in samples from dogs with EHPSS or IHPSS, 25% were not annotated. Therefore it is possible that important genes were missed because of the lack of annotation, which should be re-evaluated in the future.

A discrepancy in gene expression measured with qPCR and microarray was observed. While microarray demonstrated a significant upregulation of *HAMP* mRNA in samples from dogs with EHPSS and a significant downregulation of *HAMP* mRNA in samples from dogs with IHPSS, only the decreased *HAMP* in samples from dogs with IHPSS was confirmed by qPCR. Microarray analysis indicated a downregulation of *PALLD* RNA expression in samples from dogs with EHPSS, whereas qPCR indicated that *PALLD* was downregulated in samples from dogs with IHPSS. Similarly, *VCAM1* expression was upregulated in samples from dogs with IHPSS when measured by microarray, but downregulated when measured by PCR analysis and IHC. The use of a common reference pool containing only two control samples in the microarray study and the biological variation in the liver samples might be an explanation for these differences. In addition, the microarray is a semi-quantitative screening method, the results of which should be confirmed by qPCR and other methods. Data obtained with qPCR and protein-based assays are considered more reliable.

The expression of mRNA for cysteine conjugate Beta-lyase 1 (CCBL1) was significantly different in samples from dogs with IHPSS compared with control dogs, whereas there was no difference in samples from dogs with EHPSS after Bonferroni correction. The expression of CCBL1 protein was significantly lower, measured by immunohistochemistryand Western blot, in samples from dogs with IHPSS or EHPSS compared to samples from control dogs. Changes in *CCBL1* expression appear to be a secondary effect of portosystemic shunting, because similar differences were found in the two shunt groups compared with the control group. *CCBL1* encodes an enzyme that metabolizes cysteine conjugates of halogenated alkenes and alkanes, leading to the formation of reactive metabolites that can lead to nephro- and neurotoxicity [39]. This enzyme is probably secondarily involved in CPSS in dogs and may play a role in the pathophysiology of hepatic encephalopathy. It will be of interest to evaluate CCBL1 in diseases commonly related with hepatic encephalopathy such as cirrhosis in man and dogs.

Immunohistochemistry and Western blot confirmed the observed significant differences in the expression of *VCAM1*. In portosystemic shunting, venous blood flow to the liver is impaired, which could prompt the synthesis of angiogenic factors, in order to optimize blood supply to the liver. VCAM1 and integrin α4β1 are both involved in angiogenesis, with VCAM1 being expressed by proliferating vascular smooth muscle cells and integrin α4β1 being expressed by proliferating endothelial cells. Both integrin α4β1 and VCAM1 facilitate the adhesion of endothelial cells to vascular smooth muscle-like pericytes, which is essential for the survival of endothelial and mural cells during neovascularization. Antagonists of this integrin-ligand pair induce endothelial cell and pericyte apoptosis, thereby inhibiting angiogenesis [40]. We therefore anticipated that the expression of VCAM1 protein would be upregulated in the dogs with shunts, because a demand for angiogenic factors is to be expected due to the impaired development of the smaller branches of the portal vein tree in the liver [7]. Surprisingly, while this protein was upregulated in dogs with IHPSS, it was not in dogs with EHPSS, consistent with the qPCR findings. Given the similar physiological consequences of IHPSS and EHPSS, we suggest that the observed difference in VCAM1 expression in these two shunt types is directly related to the cause of EHPSS. In mammals the extrahepatic portal system is formed by regression of the embryonic vitelline veins [13]. Extrahepatic shunts are considered to be erroneous connections formed between the cardinal and vitelline systems during embryonic development. EHPSS could be a secondary effect of an impaired vascular remodeling of the vitelline system. Therefore the role of VCAM1 in the regression of this system needs to be further studied. The difference in qPCR results for the two different primer sets for VCAM1 was also unexpected. Both primer sets, the microarray probe, and the antibody were designed on the basis of regions of the protein present in both transcripts annotated for VCAM1 by Ensembl. The differences may indicate the presence of additional as yet not annotated transcripts in the dog. Given the function of VCAM1 in angiogenesis and the qPCR results for samples taken intraoperatively (Figure 3A), this gene or these genes involved in its regulatory pathways could be candidate genes for causing EHPSS in dogs.

The higher expression of WEE1 mRNA in samples from dogs with IHPSS measured by microarray was confirmed by qPCR analysis, immunohistochemistry and Western blot analysis. The WEE1 gene encodes a nuclear tyrosine protein kinase. In humans, WEE1 is reported to be a negative regulator of mitosis by inhibiting tyrosine 15 phosphorylation and thereby inactivating cdc2 kinase [41]. WEE1 might also have an important role in hypoxia-induced pathological processes in endothelial cells, such that its upregulation in endothelial cells under hypoxic conditions ensures cell viability [42]. Oxygen tension is known to play an essential role in the postnatal closure of a comparable structure, the ductus arteriosus [43]. Normal cardio-pulmonary adaptation after birth causes an oxygen saturation increase from 65% to more than 90% within the first minutes after birth [44]–[46]. The ductus arteriosus constricts immediately after birth, when blood oxygen tension is rising [47]. The physiological resemblance between the ductus arteriosus and the ductus venosus makes it likely that oxygen has a comparable role in the postnatal closure of these two anatomical structures. An increased expression of WEE1 might cause a protective response against altered oxygen tension, while this tension might be essential for closure of the ductus venosus as well. The owners of dogs with IHPSS did not consent to postoperative liver biopsy because of the risk and complexity of the surgical intervention. Therefore we were not able to determine expression of WEE1 after ligation of the ductus venosus and prove that it`s increase is not due to a secondary effect of the patent ductus venosus.

## Conclusions

In summary, using hepatic samples from dogs with two types of portosystemic shunt with a different genetic background but identical phenotypic consequences, we managed to identify a small list of proteins possibly involved in the two anatomical anomalies. In dogs with IHPSS, WEE1 was aberrantly over expressed, which may be related to the disturbed closure of the ductus venosus. In dogs with EHPSS, decreased VCAM1 expression may play a role in the development of intrahepatic portal vascularization. It remains to be investigated whether these proteins are directly involved in the development of portosystemic shunts, or whether they manipulate downstream genes. CCBL1 may be an interesting candidate to study unresolved factors in the pathophysiology of hepatic encephalopathy.
