# Correction: Aberrant Gene Expression in Dogs with Portosystemic Shunts

**DOI:** 10.1371/annotation/5cccaae3-f06c-413f-857e-46546e87fce1

**Published:** 2013-10-15

**Authors:** Frank G. van Steenbeek, Lindsay Van den Bossche, Guy C. M. Grinwis, Anne Kummeling, Ingrid H. M. van Gils, Marian J. A. Groot. Koerkamp, Dik van Leenen, Frank C. P. Holstege, Louis C. Penning, Jan Rothuizen, Peter A. J. Leegwater, Bart Spee

In the second to last sentence of the abstract, the first instance of IHPSS should read EHPSS. The sentence should read: "...these findings may indicate a causative role for VCAM1 in EHPSS and WEE1 for IHPSS." 

